# Two-Tiered Grading in Required Clerkships: Understanding the Why and Results of Making This Change

**DOI:** 10.7759/cureus.102579

**Published:** 2026-01-29

**Authors:** Katherine R Schafer, Jenny Wright, Andrew Caruso, Christopher J King, Susan E Merel, E Shen, Jeff LaRochelle

**Affiliations:** 1 Internal Medicine, Wake Forest University School of Medicine, Winston-Salem, USA; 2 Internal Medicine, University of Washington, Seattle, USA; 3 Internal Medicine, Baylor College of Medicine, Houston, USA; 4 Internal Medicine, University of Colorado School of Medicine, Aurora, USA; 5 Internal Medicine, University of Central Florida College of Medicine, Orlando, USA

**Keywords:** clerkships, grade reliability, grade validity, two-tiered grading, well-being

## Abstract

Background

Clinical clerkship grading has a significant and lasting impact on medical students. Recently, there has been a trend towards schools transitioning from multi-tiered to two-tiered grading in the clinical clerkships.

Objective

The goal of this study was to explore the motivation and impact of shifting to two-tiered grading in the required clinical clerkships.

Methods

The authors designed a survey to explore the motivations and impacts of using a two-tiered grading system and disseminated it to educational leaders at institutions that have transitioned from multi-tiered grading, identified through data from the Association of American Medical Colleges (AAMC).

Results

Of the 20 schools identified as utilizing two-tiered grading, curricular leaders at 11 finished the survey, and two additional schools partially completed the survey. Their responses were based on their interpretation of their own institution-specific data. Respondents noted the reliability and validity of grades and student well-being as the most important reasons for switching to a two-tiered system. After implementing two-tiered grading, student well-being and satisfaction with the grading process trended towards improvement at most institutions. Student match rate, number of students in the supplemental match, and number of residency interviews did not change. Other domains cited as reasons for the change to two-tiered grading were not tracked by institutions.

Conclusions

This study suggests that the most common motivation for transitioning to two-tiered grading for the clinical clerkship is concern about grading validity and reliability. While student well-being and satisfaction with grading trended towards improvement after changing to two-tiered grading, there was no formal monitoring by institutions of the other drivers for implementing two-tiered grading which limits further conclusions.

## Introduction

Innovation in undergraduate medical education (UME) is requisite to meet the ever-changing needs of society and to create a diverse community of physicians who are engaged and ready to care for patients. One area of potential innovation is to reconsider how medical students are evaluated in the clinical years as assessment can significantly impact residency selection, career choice, and student well-being [1,2].

Most medical schools in the United States use multi-tiered grading during their required clerkships [3]. There are several concerns with multi-tiered grading systems; they can disadvantage students of diverse backgrounds [4,5]. In addition, there are concerns about their impact on student well-being. Student well-being in the context of assessment is of particular concern given that undergraduate medical students have higher rates of depression and anxiety than other people their age and evaluations are a source of significant stress and distress [6-8].

There is significant precedent for two-tiered (also referred to as pass/fail (P/F)) grading in the preclinical years, and data suggest this approach improves student well-being without academic consequences when compared to multi-tiered grading [1,2]. More recently, there has also been an increasing trend towards two-tiered grading in the required clerkships, with 20 schools using this structure as of 2022 up from 14 schools in 2018 [3]. Notably, only 45% of schools that have two-tiered grading during required clerkships also use two-tiered grading during sub-internships, selectives, and/or electives. However, data are limited regarding the institutions' motivations to make these grading system changes and the outcomes after implementation. Existing data are largely qualitative, focused on student well-being rather than educational outcomes, and are limited to individual institutions [7-11].

A move toward two-tiered grading in both the preclinical and clinical years of UME could have unintended negative implications on learner development and the residency selection process. Longitudinal data suggest that struggling students may be disadvantaged by P/F grading as it may delay the possibility of remediation [12]. Multi-tiered grading has been used by residency programs as an important component of their selection process, with one meta-analysis finding that program directors in multiple specialties believe that core clerkship performance was a reliable representation of an applicant's preparedness for residency [13]. With the United States Medical Licensing Exam (USMLE) Step 1 moving to pass/fail, clerkship performance may be under more scrutiny as a high-stakes evaluation and may become a greater stress point for students. Residency program directors have stated their concerns about a move to two-tiered grading for the clerkship year. When reviewing applicants from institutions utilizing two-tiered clerkship grading, residency directors depend on narrative assessments more heavily, posing challenges to differentiating students and also raising the risk of biased language based on gender and underrepresented in medicine (URM) status [14]. Interestingly, surveys of residency directors have not shown a statistically significant difference in the preferential selection of students from two-tiered versus multi-tiered institutions [13]. In addition, residency selection may become more dependent on other measures such as USMLE Step 2 scores, number of research projects, or the prestige and status of the applicants' medical school [13,15]. This shift may have additional unintended consequences on students' clinical skills and overall education [16].

To build upon existing literature exploring the perspectives of students and residency directors regarding a shift to two-tiered grading, this paper describes the results of a survey of curriculum leaders at allopathic medical schools in the United States using two-tiered grading models. The survey aimed to characterize institutions' motivation for the transition to two-tiered grading, the relevant outcomes leaders are following post-implementation, and any measured impact on their students' UME to graduate medical education (GME) transition given the limited and variable data evaluating P/F grading in the clinical years.

## Materials and methods

Study design

This study used a retrospective cohort design surveying curriculum leadership at allopathic US-based medical schools using a two-tiered grading rubric for the required clerkships. The survey tool was developed by our team (as detailed below) and consisted of 15 items, many of which asked about how student performance was communicated through transcripts and the Medical Student Performance Evaluation (MSPE).

We developed a survey instrument to characterize the motivation of educational leaders in the adoption of a two-tiered grading system for core clerkships as well as the perceived and measured (when available) impacts of that change in the following categories of motivators and impacts: reliability and validity of assessments, fairness and bias of assessments, student well-being, impact of competency-based education, and external and internal pressures. Based on literature suggesting the benefits and strengths of two-tiered grading, we chose some specific elements within these categories including average USMLE Step 2 scores, post-graduate year 1 (PGY1) feedback from residency programs, student satisfaction in the grading system, student engagement in clinical education, residency interview invitation numbers (for all students and URM students), number of students in the supplemental match, and overall residency match rates (for all students and URM students) [4-8]. Respondents were not given a strict p-value definition for "statistical improvement"; this distinction relied on the respondent's internal data analysis standards.

Data collection methods

To ensure the clarity, relevance, and comprehensiveness of the survey instrument, we conducted expert validation and pilot testing. Academic leaders and subject matter experts from several of the authors' affiliated institutions reviewed the survey prior to dissemination. Their feedback was used to assess whether the items effectively captured all key dimensions of the primary constructs under investigation. This iterative process helped refine item wording, improve construct coverage, and ensure the instrument's overall content validity prior to full deployment to target institutions. To mitigate bias in responses, items were phrased as direct questions to leadership regarding institutional memory and perception with balanced response scales matched to item questions. Response scales were standardized across the survey, and the survey was anonymous.

Sample characteristics

We obtained data from the Association of American Medical Colleges (AAMC) in June 2023 to identify our study population: allopathic US medical schools used two-tiered grading in the required clerkship years. We applied a non-probability sampling strategy. Schools were included if they used two-tiered grading in the required clerkship years. Two investigators (AC, CK) researched the leadership structure of these schools and identified the leader most likely to have spearheaded this grading change (e.g., Associate Dean for Curriculum or Assessment). Survey invitations were sent to all 20 schools that used a two-tiered grading system during the clerkship years.

Survey administration

The study team then contacted each institution's designated leader with a standard email inviting them to complete the online survey via the Qualtrics survey software (Provo, Utah, United States). Only one individual from each institution completed the survey. Biweekly reminders were sent until the survey was closed approximately five weeks after the initial invitation. Survey responses were anonymous. Full text of the survey is available upon request.

Ethical considerations

All study procedures and disseminated materials (survey, email language) were approved by the Institutional Review Boards of the Wake Forest University School of Medicine and the University of Central Florida, which deemed the study "Exempt" due to the lack of personal health information.

Statistical analysis

Results of the survey were analyzed using IBM SPSS Statistics for Windows, Version 27.0 (IBM Corp., Armonk, New York, United States), for descriptive statistics.

## Results

**Figure 1:**
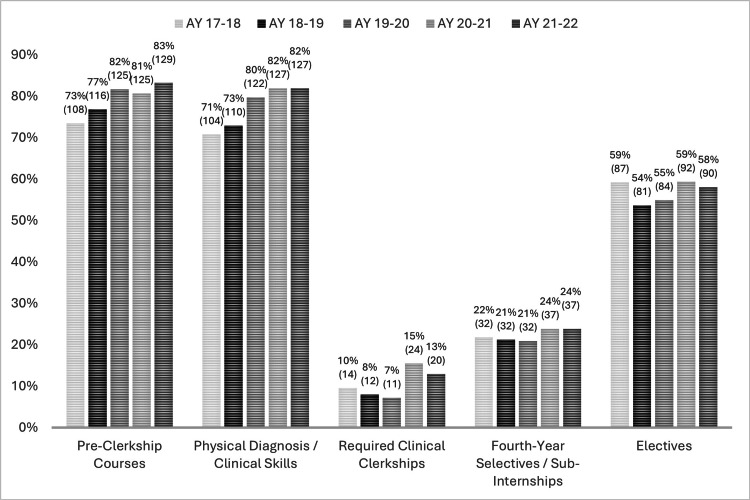
Trends in grading: AAMC data reflecting the use of two-tiered grading across different phases of medical school (n=155 AY21-22 and AY20-21; n=152 AY19-20; n=151 AY18-19; n=147 AY17-18) AY: academic year; AAMC: Association of American Medical Colleges

Twenty out of 155 (12.9%) schools used P/F grading in the required clerkships for the academic year 2021-2022 (Figure 1). The overall survey response rate was 65% (among 20 schools invited, only 13 completed the survey), though two schools did not answer all questions. We considered the missing data as Missing at Random (MAR). For the existing response data set, we analyzed it on a question-by-question basis when data was available. We are unable to disclose the names of the individual institutions per AAMC data policy, but many are considered "top-tier" institutions in the US allopathic medical school community (e.g., highly ranked in widely accepted national polls). The schools both invited to and that completed the survey were from diverse geographic regions across the continental United States.

Table 1 shows the factors that institutions used to decide to move to a P/F core clerkship, with the three most important factors being reliability of grades in the old system, validity of grades in the old system, and student well-being. Similarly, when asked to describe the relative importance of each factor in the decision to change to a P/F clerkship, respondents selected fairness (81.8%), validity (81.8%), reliability (71.7%), and student well-being (54.6%) as either "extremely important" or "very important" (Table 2).

**Table 1:** Prioritized factors involved in the decision to move to two-tiered grading: Responses to "Please rank the three most important factors that went into the decision to move to P/F core clerkships (drag and drop items in rank order)" (n=13) Ranking: 1: most important; 10: least important; "other – please specify" choice is not included in the table. LCME: Liaison Committee on Medical Education; P/F: pass/fail

Prioritized factors involved in the decision to move to two-tiered grading	Average ranking
Reliability (consistency and reproducibility) of grades in the old system	3.5
Validity (accuracy and defensibility) of grades in the old system	3.5
Student well-being	3.5
Fairness in grading	3.7
Transition to competency-based medical education	4.6
Student satisfaction with the old grading system	5.0
LCME accreditation	7.5
National trends	8.1
Grade appeals	8.2

**Table 2:** Rating of the importance of factors in the decision to change to a P/F required clerkship (n=11) P/F: pass/fail

	Extremely or very	Extremely	Very	Moderately	Slightly	Not at all
Validity (accuracy and defensibility) of grade decisions	81.8%	54.5%	27.3%	18.2%	0%	0%
Concerns over fairness in grade decisions	81.8%	72.7%	9.1%	9.1%	9.1%	0%
Reliability (consistency and reproducibility) of grade decisions	72.7%	54.5%	18.2%	18.2%	9.1%	0%
Student well-being	54.6%	27.3%	27.3%	27.3%	18.2%	0%
Transition to competency-based medical education	45.5%	27.3%	18.2%	27.3%	9.1%	18.2%
Consequences of clerkship grades on residency choice	27.3%	9.1%	18.2%	36.4%	18.2%	18.2%
National trends	18.2%	0%	18.2%	18.2%	0%	63.6%
Student satisfaction with grading system	9.1%	0%	9.1%	54.5%	27.3%	9.1%
Institutional pressure	0%	0%	0%	18.2%	18.2%	63.6%
Grade appeals	0%	0%	0%	9.1%	36.4%	54.5%

When asked to report the actual impact of the change to a P/F core clerkship at their institution, respondents were asked to report "statistical" improvement/worsening, "trends towards" improvement/worsening, "no change", "did not formally assess", or "do not know". Combining these outcomes into four groups, namely, improvement ("statistical" and "trend towards" improvement), no change, worsening ("statistical" and "trend towards" worsening), and did not assess or know, revealed improvement in several measures including student well-being (63.6%), student satisfaction (63.6%), reliability (45.5%), and validity (45.5%). Around 9.1% of respondents reported worsening in average USMLE Step 2 Clinical Knowledge (CK) scores, PGY1 feedback from residency programs, and student engagement in clinical education (Table 3). Nearly 55% of respondents "Did not assess" URM match data (Table 3).

**Table 3:** Schools' reported impact of the change to a P/F core clerkship (n=11) *: combined results for "statistical improvement" and "trend towards improvement" **: combined results for "trend towards worsening" and "statistical worsening" USMLE: United States Medical Licensing Exam; CK: Clinical Knowledge; PGY1: post-graduate year 1; URM: underrepresented in medicine

	Improvement*	No change	Worsening**	Did not assess or do not know
Average USMLE Step 2 CK scores	18.2%	54.5%	9.1%	18.2%
PGY1 feedback from resident programs	18.2%	45.5%	9.1%	27.3%
Student well-being	63.6%	27.3%	0%	9.1%
Student satisfaction in the grading system	63.6%	27.3%	0%	9.1%
Student engagement in clinical education	27.3%	54.5%	9.1%	9.1%
Reliability (consistency and reproducibility) of grade decisions for P/F	45.5%	27.3%	0%	27.3%
Validity (accuracy and defensibility) of grade decisions for P/F	45.5%	27.3%	0%	27.3%
Student perceived fairness in preceptor evaluations	45.5%	36.4%	0%	18.2%
Number of residency interviews for all students	0%	72.7%	0%	27.3%
Total number of students in the supplemental match	0%	81.8%	0%	18.2%
Overall residency match rate	0%	81.8%	0%	18.2%
Number of residency interviews for URM students	9.1%	36.4%	0%	54.6%
Number of URM students in the supplemental match	0%	45.5%	0%	54.6%
Residency match rate for URM students	9.1%	45.5%	0%	45.5%

**Figure 2:**
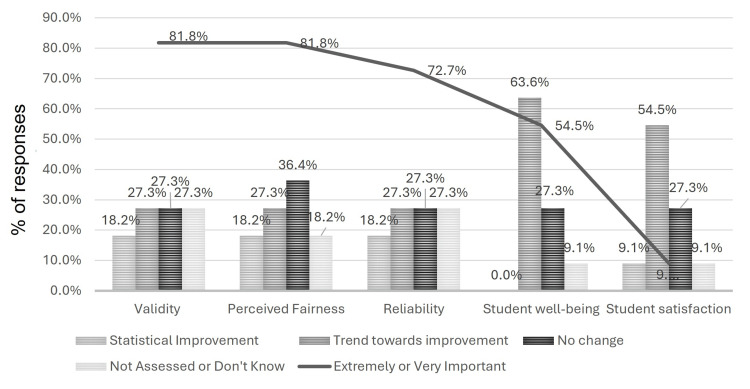
Comparison of improvement in outcomes (bar graph) to the relative importance of the outcome as a motivation to transition from multi-tiered to two-tiered grading (linear graph)

Other than student satisfaction, all factors noted as changing after the implementation of a P/F grading system were highly rated as motivations for the grading change. Figure 2 shows the factors that improved after the implementation of a P/F grading in relation to the importance of the factors in the decision to make the change. Schools were not asked to define "statistical improvement" versus "trend toward improvement".

## Discussion

As of the academic year 2021-2022, 20 US allopathic medical schools used P/F grading for the required clerkships. Additional schools have adopted this approach in 2023-2025, suggesting a continued trend towards embracing binary grading practices (KRS, personal communication).

Leaders at over a quarter of programs did not know, did not collect data, or had not yet assessed if the move to two-tiered grading impacted the reliability and validity of grading, factors that were deemed highly important in motivating the transition from multi- to two-tiered grading. Very few institutions reported statistical improvement in these factors with most reporting trends towards improvement, suggesting the intended impact of this grading change has not been realized or systematically measured. Rigorous studies of the transition to two-tiered grading are essential to determine the most reliable, valid, and equitable grading practices while also measuring changes in other important psychosocial parameters. Thoughtful review of student assessments for their reliability and comparisons against a standard, perhaps in an objective structured clinical exam (OSCE) setting, to measure validity might also be valuable. 

Around 9.1% of respondents reported worsening in average USMLE Step 2 CK scores, PGY1 feedback from residency programs, and student engagement in clinical education. While this is not a majority, because assessment often drives learning and performance, this signal of lower student engagement and testing performance is concerning and should be followed over time. 

If the motivation to move towards P/F grading is to address fairness, inequities in grading, or student well-being, the fact that most schools that have two-tiered grading in the required clerkships have multi-tiered grades for the sub-internships/selectives and electives creates an inconsistent approach. These issues may be compounded by compressing the students' ability to differentiate themselves in courses with multi-tiered grading into fewer clinical rotations with fewer assessment opportunities leading to increased issues regarding validity [17]. Furthermore, the majority of respondents "Did not formally assess" URM match data, which is concerning given that "fairness" was cited as highly important in motivating schools to change their grading systems. Follow-up studies are needed to explore the effect the transition to two-tiered grading has on student well-being, its relationship with goal orientation (mastery vs. performance), and concerns regarding grade reliability in sub-internships, selectives, and clinical electives.

It is worth noting that many schools adopting P/F grading and surveyed here are perceived to be highly prestigious. Schools that benefit from higher prestige may not experience any negative effect on their students' residency interview or match rate due to the benefit of their brand. It is unclear whether the effect of moving to P/F grading at less prominent institutions would be similar in the absence of a prestigious name. In a recent survey of residency program directors, the majority had concerns about clerkships transitioning to P/F grading yet also reported that P/F grading did not make applicants less likely to be selected for their residency program. In the absence of multi-tiered grading, they reported being more reliant on other measures, including a school's prestige [13]. This finding raises concern that the grading change, which was in part made to address inequities, may increase other types of inequities, making it harder for a high-achieving student from a less prestigious medical school to be selected for a more competitive residency program. 

Our study has several limitations. First, this survey of institutional leaders is limited to a discussion of communication in student transcripts and in the MSPE letter. Future investigations should include how information about student performance is communicated in Structured Evaluative Letters (SEL) or Department Letters used in residency applications for internal medicine, pediatrics, and emergency medicine residencies. It is unclear if schools are eliminating tiered grading, but continuing to stratify students through the use of summary adjectives in the MSPE, SEL, or Department Letter. Notably, our survey did not ask for the actual data from schools on the parameters of interest; rather, it asked for an official's report of the data as an initial exploratory investigation. As such, recall and social desirability biases could have affected some of the survey item responses. A follow-up study could include a request from each school for their data on the categories of interest, though whether or not they would be willing to participate could create further limitations. Some schools may not have had enough time to collect outcome data since changing their required clerkship grading to P/F. Additionally, while some research suggests that two-tiered grading encourages a mastery-oriented learner mindset and, in turn, supports long-term learner development, our survey did not explore schools' specific processes for the determination of grades; therefore, we do not know how changing to two-tiered systems might have altered their actual grading process [7,18]. Lastly, given the lack of multi-center investigations of two-tiered grading, we cannot draw comprehensive comparisons with our data. As such, this study's utility is the identification of knowledge and measurement gaps for this curriculum change.

## Conclusions

Based on a retrospective survey of educational leaders at a small group of schools that are currently using two-tiered or P/F grading, the most common motivation for transitioning to two-tiered grading for the clinical clerkship is concern about grading validity and reliability. Student well-being and grading validity are perceived to be improved by school leadership, but there has been little formal tracking of these measures after a change to P/F grading. There are insufficient data to support a "passing grade" for all institutions to adopt the practice at this time. More data and formal institutional assessments are needed before universally changing clerkship grading to P/F. Perhaps the question we should be asking as we educate future physicians is not just how to improve well-being and reduce stress, but how to more effectively manage and distribute the stress across the UME to GME continuum.
